# Manufacture of Reduced Fat White-Brined Cheese with the Addition of β-Glucans Biobased Polysaccharides as Textural Properties Improvements

**DOI:** 10.3390/polym12112647

**Published:** 2020-11-10

**Authors:** Efthymia Kondyli, Eleni C. Pappa, Alexandra Kremmyda, Dimitris Arapoglou, Maria Metafa, Christos Eliopoulos, Cleanthes Israilides

**Affiliations:** 1 Dairy Research Department, Institute of Technology of Agricultural Products, Hellenic Agricultural Organization-DEMETER, Katsikas, 45221 Ioannina, Greece; pappa.eleni@yahoo.gr; 2 Division of Food, Nutrition and Dietetics, School of Biosciences, University of Nottingham, Sutton Bonington Campus, Loughborough, Leicestershire LE12 5RD, UK; Alexandra.Kremmyda2@nottingham.ac.uk; 3 Institute of Technology of Agricultural Products, Hellenic Agricultural Organisation-DEMETER, 1 S.Venizelou, 14123 Lycovrysi, Greece; dimarap@yahoo.com (D.A.); mariametafa@gmail.com (M.M.); chris_eliopoulos@hotmail.com (C.E.); cisrailides@yahoo.gr (C.I.)

**Keywords:** white-brined, cheese, β-glucan, mushroom, low-fat

## Abstract

β-Glucan, isolated from the mushroom *Pleurotus ostreatus*, at a concentration of 0.4%, was used in the manufacture of reduced-fat white-brined cheese from sheep milk. Control reduced-fat cheese was also produced from the same milk without the addition of β-glucan. The resultant cheeses were examined for their physicochemical characteristics, color and textural properties, and level of proteolysis and lipolysis. Furthermore, cheeses were evaluated organoleptically. In general, there were no statistical differences in the physicochemical characteristics and proteolysis levels found between both cheeses. The addition of β-glucan improved textural properties, and the cheeses received favorable grades for all the organoleptic characteristics. There were no flavor defects (such as a bitter taste) described by the panellists in this study. Generally, the addition of β-glucan did not significantly affect total free fatty acid content; however, at 180 days of ripening and storage, cheeses with the addition of β-glucan had a higher (*p* < 0.05) content than cheeses without β-glucan. The major fatty acids were acetic acid and capric acid.

## 1. Introduction

Dietary fat, and particularly specific saturated fatty acids, can be associated with an increased risk of many diseases, such as obesity, atherosclerosis, coronary heart disease, etc.; therefore, the current trend in nutrition and health awareness leads consumers to low-fat food products, including low-fat cheeses. Furthermore, consumers expect that low-fat cheeses will be almost identical to full-fat ones in their physicochemical and sensory attributes. Usually, the main undesirable characteristics of low-fat cheeses are that they can be too firm, and have a harder and more rubbery texture and inferior taste compared to their full-fat counterparts [1,2,3]. Therefore, the challenge in low-fat cheese development is to improve both its sensory attributes and textural properties in order to produce a cheese that is comparable to the full-fat product [4].

To overcome the defects of low-fat cheeses, various suggestions have been made, such as the addition of specific adjunct cultures [1,3]. Another suggestion to compensate the drawbacks in texture and sensory-related attributes is to use fat replacers [1], such as β-glucan.

β-Glucan is a polysaccharide of D-glucose monomers linked by (1→3), (1→4) or (1→6) β-glycosidic bonds, originating from plants (such as oat, barley) or fungus. Usually, β-glucan is water-soluble and has the ability to form highly viscous solutions. This characteristic is a desirable attribute in the industrial processing of low-fat dairy food products, such as yoghurt and cheese. Lazaridou and Biliaderis [5] reported that β-glucan imparts moisture retention and modifies the texture and appearance of food products.

Attention has been focused on the potential use of β-glucan from oats and other cereals as a functional ingredient. β-Glucan is considered to be a functional bioactive ingredient which promotes several health benefits, such as the reduction of cholesterol levels and the reduction of postprandial glycemic and insulin response [6,7,8]. The Food and Drug Administration [9] has approved the claim regarding the cholesterol-lowering effect of β-glucan at a level of 3 g/day. The use of β-glucans from other plant sources, i.e., mushrooms and yeast, has also shown similar effects [10]. It is known that *Pleurotus* mushrooms contain numerous therapeutic substances and can provide health benefits, and have been used in antitumor, immunomodulation, anti-diabetic, cardiovascular and even cancer treatments [11]. Mushrooms are also currently considered as functional foods due to their natural ability to accumulate various types of substances that improve their health-promoting properties and can supplement the human diet [12,13]. The β-glucans contained in oyster mushrooms have been shown clinically to possess immuno-stimulating properties; among them, the best tested is pleuran, isolated from *P. ostreatus* [14].

The addition of β-glucan has been reported in yogurts [15,16,17,18,19], cream cheese [20], cheddar cheese [21,22], Kashar cheese [23] and milk beverages [24]. The fortification of a low-fat white-brined cheese from bovine milk with a commercial oat β-glucan concentrate has also been reported before [2].

The objective of the present study was to investigate the addition of β-glucan isolated from the mushroom *Pleurotus ostreatus* in the manufacture of a low-fat white-brined cheese from sheep milk.

## 2. Materials and Methods

### 2.1. Isolation and Measurements of β-Glucan from Mushrooms Pleurotus ostreatus

β-Glucans were isolated according to Wang and Zhang [25], with some modifications, from *Pleurotus ostreatus* mushrooms. Briefly, the fruiting body of the mushroom was frozen (−20 °C), cut to pieces, freeze dried (−75 °C for 24 h), lyophilized until a constant weight and powdered. Then the powder of the mushroom was defatted with ethanol by using a Soxhlet extractor for 8 h and the resultant residue was immersed in 0.9% NaCl solution at 70 °C for 24 h, centrifuged at 5700 rpm for 10 min to remove the water-soluble polysaccharide, and the residue was extracted with 1 M NaOH at 40 °C for 8 h. The supernatant was then neutralized by 1 M CH_3_COOH and the precipitate(β-glucan) was collected and washed with distilled water several times to achieve bleaching. β-Glucan in this stage is referred as β-glucan in the form of paste.

Total glucans were hydrolyzed by using the enzymes exo,1,3-β-glucanase and β-glucanase; followed by the measurement of glucose molecules. α-Glucans were hydrolyzed by the enzymes amyloglucosidase and α-amylase. β-Glucan was measured using the following equation: β-glucan = (total glucan + oligomers) − (α-glucan + oligomers) [26].

### 2.2. Structure of β-Glucan and Molecular Weight Determination

The structural and purity characteristicsofβ-glucan isolated from the *Pleurotus ostreatus* mushroom have been studied using solid state CPMAS (cross polarization magic angle spinning) NMR, [27]. Solid state NMR is a favored analysis method due to issues surrounding the solubility of β-glucan fractions.

^13^C CPMAS NMR spectra were recorded on a Bruker AVANCE III 600 NMR spectrometer (Karlsruhe, Germany) with a narrow bore magnet and 4 mm triple resonance probe. The samples were packed into 4 mm rotors and spun at 10 kHz. Chemical shift (ppm) scales were referenced to the up field peak of adamantane (29.5 ppm) run as an external standard under identical conditions. For all CPMAS experiments a contact time of 2 msec. was used. This contact time was close to the maximum signal intensity generation as determined by a variable contact time experiment. In total, 2048 scans were recorded and an exponential line broadening of 15 Hz was applied. The presence of β-glucan is conventionally indicated by the carbon 1 signal having a peak chemical shift value of approximately 104 ppm [27].

The extracted beta-glucan samples were characterized using size-exclusion chromatography (SEC) equipped with multiangle light scattering (MALS) (Wyatt Technology Corporation, Santa Barbara, CA, USA), as described elsewhere [28,29]. The machine was filled with a light-scattering instrument (DAWN ^®^HELEOS™ 18 angle light scattering photometer), which included a K5 cell type having a wavelength of 658.6 nm and was calibrated with the constant of 3.9220 × 10^−5^ 1/(V cm). Moreover, the machine was connected with an refractive index (RI) director (Optilab^®^T-REX, Wyatt Technology Corporation), a Viscometer (ViscoStar^®^) and a UV instrument (Shimadzu SPD-6AV) (Wyatt Technology Corporation). The software of ASTRA™ (Version 6) (Wyatt Technology Corporation) was used to estimate the average molecular weight (MW).

### 2.3. Cheese Manufacture

Three cheesemaking trials were carried out at the pilot plant of the Dairy Research Department according to the following procedure, which is based on feta cheese manufacturing. Fresh raw ewe milk was standardized to 3% fat content and β-glucan isolated from *Pleurotous ostreatus* in the form of paste was first dispersed in a small quantity of reduced fat milk; this was then added to the rest of the milk in a quantity of 0.4 g glucan/100 g cheese. The milk was pasteurized at 63 °C for 30 min and then cooled at 37 °C. At that temperature, a freeze-dried, direct to vat set (DVS) starter culture (FRC-60; Chr. Hansen) consisting of *Streptococcus thermophilus* and *Lactobacillus delbrueckii subsp. bulgaricus*, 1:1, was used following the supplier’s instructions. To assist the curdling of milk, CaCl_2_ (0.01%) and powdered calf rennet (HALA, Hansen’s Laboratorium, Copenhagen, Denmark) were added at 35 °C, to achieve curdling in approximately 50 min. The cheese curd was cut into small 2.5 cm cubes which were allowed to rest for 10 min and then transferred into rectangular molds. After draining (16–18 °C for 20 h), the curd was cut into blocks weighing about 1.5 kg and put into tin vessels. Granular recrystallized NaCl was added (2.2%) and after one day the drained whey was removed and replaced by 7% NaCl solution. The tin vessels were sealed and left for ripening at 16–18 °C for approximately 15 days. They were then transferred into the cold storage rooms (3–4 °C) where they remained for up to six months.

A control cheese was also manufactured from the same sheep milk without the addition of β-glucan.

### 2.4. Physicochemical Analyses

The cheese was examined at 1, 15, 30, 60, 90, 120 and 180 days of ripening and storage for pH electrometrically (Micro pH 2002; Crison, Barcelona, Spain) and was analyzed for fat content by the Gerber-Van Gulik method [30], salt content by the modified Volhard method [31], salt in moisture content by the formula SM% = S × 100/M (where S: % NaCl of cheese, and M: % moisture of cheese [32]), ash content by the method described by the International Dairy Federation [33], moisture content by drying the cheese to a constant weight at 105 °C [34], cheese acidity by the Association of Official Analytical Chemists standard method [35], and water activity (aw) using the Novasina unit Thermoconstanter, HamidatTH-2/RTD-33/BS (Novasina AG, Zurich, Switzerland). Yield was expressed in kg of cheese produced from 100 kg of milk. The moisture in non-fat substance (MNFS) content was calculated by the formula MNFS% = M × 100/100 − F, and the fat in dry matter (FDM) by the formula FDM% = F × 100/(100 − M) (where F: % fat of cheese, and M: % moisture of cheese).

### 2.5. Textural Analyses

The textural properties of mature (60, 90, 120, 180 days) cheese were analyzed using an Instron, Universal Testing Instruments Model 1011 (Instron Ltd., High Wycombe, Bucks, UK) equipped with a 50 N load cell. The cheese samples had 20 mm × 20 mm × 20 mm dimensions, were placed on a small dish, covered with an airtight plastic-wrap adhesive membrane, and allowed to equilibrate to the measuring temperature (20 °C). They were then compressed to 70% of their original height with a plunger speed of 30 mm/min. From the compression curves, the following textural characteristics were calculated: (a) brittleness or fracturability (kg), as the force to fracture the cheese sample, (b) hardness (kg),as the force recorded at 70% compression of the sample, and (c) the compression (%) at which the sample fractured. Seven replicate measurements on each cheese sample were made and the average values for the three cheesemaking trials are reported.

### 2.6. Colour Measurements

The color examination of cheese samples at 1, 15, 30, 60, 90, 120 and 180 days of ripening and storage was performed using a Hunter Lab DP-9000 (Hunter Associates Laboratory, Inc., Reston, VA, USA) colorimeter. The L*, a*, and b* color parameters were determined according to the CIELAB color space, i.e., L* corresponds to light/dark chromaticity (changing from 0% dark to 100% light), a* to green/red chromaticity (changing from 60% green to 60% red), and b* to blue/yellow chromaticity (changing from 60% blue to 60% yellow). The instrument was calibrated with a black and a white tile before the measurements.

### 2.7. Organoleptic Evaluation

The organoleptic evaluation of cheese after 60, 120, and 180 d of ripening was carried out by a five-member trained panel familiar with feta cheese. The panel was asked to evaluate the appearance, body-texture and flavor, and to notice any defects, according to the International Dairy Federation [36] guide for the organoleptic evaluation of the cheese. All these attributes were graded on a 0–10 point scale (0 = lowest quality, 10 = best quality). More importance was given to body and texture and to flavor than to appearance. Thus, the scores obtained for these two attributes were multiplied by 4 and 5, respectively, while for appearance by 1. The total score was obtained by adding the scores of the three attributes. An excellent cheese obtained a total score of 100.

### 2.8. Proteolysis

Proteolysis was assessed by measuring different nitrogen fractions. Cheese samples at 1, 15, 30, 60, 90, 120 and 180 days of ripening and storage were analyzed for total nitrogen (TN) and soluble nitrogen (SN) fractions, i.e., water-soluble nitrogen (WSN), and nitrogen soluble in 12% trichloroacetic acid (TCA-N) and in 5% phosphotungstic acid (PTA-N).Total nitrogen (TN) was determined by the Kjeldahl method [37], and water-soluble nitrogen (WSN) and nitrogen soluble in 12% TCA (TCA-SN) were determined by the Kjeldahl method on fractions of the same cheese prepared as described by Kuchroo and Fox [38], except that a Sorvall Omni-mixer (Dupont Company, Newton, CT, USA) was used for homogenization and the supernatant obtained was filtered through No. 42 filter paper. Nitrogen soluble in 5% phosphotungstic acid (PTA-SN) was determined by the Kjeldahl method according to Stadhouders [39], except that the cheese extract was prepared as described above.

### 2.9. Lipolysis

Free fatty acids of cheese samples at 1, 15, 30, 60, 90, 120 and 180 days of ripening and storage were extracted following the method as described by De Jong and Badings [40]. A Shimadzu model GC-17A gas chromatograph (Shimadzu Scientific Instruments Inc., Columbia, MD, USA), equipped with an on-column injector and a flame ionization detector (FID), was used. The column used was SGE, BP21-FFAP (15 m × 0.53 mm × 0.5 μmi.d.). Total free fatty acids (TFFA) were determined by the addition of the concentrations of the free fatty acids (FFA).

### 2.10. Statistical Analysis

The data were analyzed by one-way analysis of variance (ANOVA), performed using the software Statgraphics (Statistical Graphics Corp., Rockville, MD, USA). When significant (*p* < 0.05) differences were found among cheeses with or without the addition of β-glucan, the means were separated by a least significant difference test (LSD).

## 3. Results and Discussion

### 3.1. Structure and Molecular Measurements of β-Glucan from Mushrooms Pleurotus ostreatus

Edible mushrooms could be an excellent source of bioactive carbohydrates, including β-D-glucans. The β-D-Glucans of mushrooms present variability in the way of linkages, depending on the fungal species [41]. The *Pleurotus ostreatus* mushroom contains glucans with a backbone structure including (1→3)-links every fourth residue, which are substituted at O-6 with single D-glucopyranosyl groups [42]. However, different structural patterns of polysaccharides from the *Pleurotus* species have been observed. For example, both a linear α-(1→3)-linked D-glucan and a β-(1→3), (1→6)-linked glucan have been isolated from *Pleurotus ostreatus* [43].

**Figure 1:**
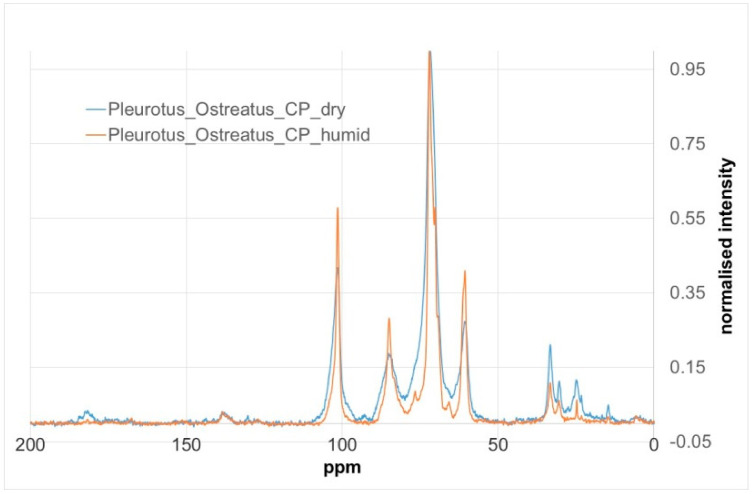
Cross Polarization/Magic Angle Spinning Nuclear Magnetic Resonance (CPMAS NMR) spectra of β-glucan from *Pleurotus ostreatus*.

The CPMAS NMR results of an extracted β-glucan sample from a wild sample of *Pleurotus ostreatus* mushroom can be observed in Figure 1. The spectrum showed a broad peak from less than 100 to greater than 104 ppm, which is characteristic of both α- and β-glucans. The extracted β-glucan sample was also subjected to membrane dialysis and hydration in order to reduce the amount of sodium acetate formed in the extraction process. However, the two samples, *Pleurotus ostreatus* and *Pleurotus ostreatus* humid, did not present any fundamental differences in their CPMAS NMR spectra, the humidified sample merely showing narrower peaks. Moreover, the β-glucan content, measured using the enzymatic method of MEGAZYME, showed that the content of the *Pleurotusostreatus* was 0.53% w/w and 3.1% w/w in dry matter, which is much lower than in the results of other studies [44,45].

Humidification is a way of inducing structural stabilization, i.e., allowing the chemical structure to reach a lower energy and a more stable configuration, which results in sharper peaks and easier assignment. It has the added benefit of dissolving and reducing any soluble salts remaining from the extraction procedure.

The average molecular weight (MW) of the β-glucans extracted from the mushroom *Pleurotus ostreatus* was between 3.09 and 5.00 kDa (average 4.00 kDa). The β-glucans of the mushrooms are lower MW compounds in comparison to the β-glucans originating from plant sources or algae. It has been suggested that the high MW and extensive branching points of the β-glucan molecule increase the immunostimulatory and immunoregulatory effect. Nowadays, it is well established that both the immunostimulatory and immunoregulatory effects of the β-glucans are independent of the MW of the β-glucan molecule, which is of great importance in supporting the immunostimulatory and immunoregulatory effect of small MW β-glucans, and their potential effect as biomedicines in the future [46].

The quantity of β-glucan that was added during the cheese-making was 0.4 g/100 g of cheese. From Table 1 it can be seen that this quantity, as measured using the Megazyme method [26], remained stable in the cheese mass at all sampling days except at the 1st day, i.e., after curdling. At that age, the quantity of β-glucan was higher (0.75%) than the estimated added quantity (0.4%), and this can be attributed to the quantification method used in this study. The Megazyme method used [26] measured the glucose molecules from the lactose found in cheese samples. At the first day of ripening, the lactose was not yet fermented to lactic acid, therefore with this method the quantity of lactose remaining in the cheese was taken into account, while from the 15th day onwards, the lactose in the cheese samples was converted to lactic acid.

### 3.2. Physicochemical Analyses

The physicochemical characteristics of white-brined cheese produced with (G) or without (C) the addition of β-glucan, at different sampling dates, are shown in Table 2 and Table 3. It can be seen that, generally, there were no statistical differences found between the G or C cheeses. At the 1st and 180th day of ripening and storage, the moisture and the moisture in non-fat substance contents of G cheeses were higher (*p* < 0.05) than those of C cheeses. Furthermore, at day 1, the G cheese showed lower (*p* < 0.05) acidity content than the C cheese, and at the 30th day the G cheeses had higher (*p* < 0.05) yields than the C cheeses.

The pH values of the white-brined cheeses, regardless of the addition of β-glucan, decreased until they entered the cold room (15 days). Afterwards, the pH remained at a stabilized level in the range of 4.51–4.36 for both cheeses, securing a good keeping quality [47,48]. In general, similar pH values were reported for other cheeses of the same variety [2,49,50,51,52].

The moisture content was 69.04–70.68% at the 1st day, and decreased to 63.31–63.35% when transferred to the cold room (15th day). The decrease in moisture, as well as the pH, is due to the lactose fermentation and the consequent acid development, as well as to the syneresis of cheeses. Other contributing factors may be the reduced hydration of casein as the pH reaches its isoelectric point, and the ability of NaCl to create high osmotic pressure and the consequent release of moisture from cheese into brine [53]. The moisture in non-fat substance (MNFS) ranged between 77.38–76.14% at the 1st day of ripening and storage and 72.0–74.06% at the other sampling dates. Volidakis et al. [2] observed higher moisture levels, but Katsiari and Voutsinas [54] found lower moisture and MNFS contents than in the cheeses without the addition of β-glucan in this study. The above differences could be attributed to the differences in milk composition and the different manufacturing conditions used.

The mean values of fat, fat in dry matter (FDM), salt, salt in moisture (SM), ash and water activity (aw) of the cheeses manufactured with or without the addition of β-glucan did not differ significantly (*p* > 0.05) at all sampling dates (Table 2 and Table 3). Higher values were found by Katsiari and Voutsinas [54] in sheep low-fat feta-type cheese made with 3% fat compared to the cheeses of this study. Volidakis et al. [2] observed lower fat but higher salt contents in low-fat white-brined cheeses made with cow milk. The different types of milk, production methods and ripening conditions may explain the above differences.

The cheese yield values of low-fat white-brined cheese in this study were in accordance with the results of Katsiari and Voutsinas [54].

### 3.3. Textural Analyses

The results of the textural properties of mature, i.e., after 60 days of ripening and storage, white-brined cheeses made with (G) or without (C) the addition of β-glucan, during storage, are shown in Table 4.

From Table 4 it can be seen that, until the 120th day of ripening and storage, cheeses manufactured with the addition of β-glucan showed lower (*p* < 0.05) values of hardness and brittleness than cheeses made without the addition of β-glucan (control). At the age of 180 days, no significant differences were observed between C and G cheeses. The compression to fracture values did not differ significantly at all sampling dates. The addition of β-glucan improved the textural properties of the white-brined cheeses, generally.

### 3.4. Colour Measurements

Color is an important quality parameter, which together with flavor affects the consumer preference. Concerning color parameters (Table 5), the addition of 0.4% of β-glucan did not significantly (*p* > 0.05) affect the cheese samples except at day 120, in which the C cheeses showed higher values than the G cheeses, for the color parameter a*. Therefore, in general, cheeses with (G) or without (C) β-glucan showed the same luminous (parameter L*) and yellow (parameter b*)–green (parameter a*) color. The values of the L*, b* and a* parameters of this study were within the range of the white color.

### 3.5. Organoleptic Evaluation

The organoleptic properties of white-brined cheeses made with or without the addition of β-glucan are shown in Table 6.

From the production method used in this study, both G and C cheeses received very high scores for all the organoleptic characteristics, as shown in Table 6. There were no statistical differences between G and C cheeses, except that at 120 days of ripening and storage the cheeses with G received higher flavor grades than control cheeses. Therefore, the addition of 0.4% of β-glucan into the cheese did not affect its organoleptic properties, and was very much accepted by the panellists.

### 3.6. Proteolysis

The rate and extent of proteolysis in the cheeses, monitored by measurements in the levels of WSN, TCA-N and PTA-N produced during ripening and storage, are shown in Table 7. All soluble fractions increased in both the G and C cheeses throughout ageing.

No statistically significant differences were observed in the soluble factions of C and G cheeses, generally. However, G cheeses showed, at 90 days of ripening and storage, lower WSN levels, and at 120 days lower TN% and proteins contents than C cheeses (Table 7). In this study, the proteolysis of low-fat cheeses was lower than that of feta cheese [51] and of low-fat white-brined cheeses [2,54]. Katsiari and Voutsinas [54] also reported that low-fat cheeses had lower proteolysis levels than full-fat cheeses.

### 3.7. Lipolysis

The evolutions of total FFAs (TFFAs), individual FFAs and acetic acid in low-fat white-brined cheese made with or without the addition of β-glucan, at different sampling days, are presented in Table 8 and Table 9. Fatty acids come mainly from lipolysis (hydrolysis of triglycerides into free fatty acids) during the ripening of cheese. Generally, the addition of β-glucan did not significantly affect TFFA content except at 180 days of ripening and storage, the date at which G cheeses showed higher (*p* < 0.05) contents than C cheeses (Table 8 and Table 9).

The major fatty acids were acetic acid (C2) and C10 (capric acid). Acetic acid is not a product of lipolysis; it is mainly a product of other biochemical pathways, such as the fermentation of lactic acid, and it characterizes, generally, white-brined cheeses with a harsh but not rancid flavor, and a related typical aroma [47]. High levels of acetic acid were found in Teleme and feta white-brined cheese [55,56]. Capric acid was the dominant free fatty acid in Urfa ovine white-brined cheese [57].

## 4. Conclusions

Since some forms of dietary fat are linked to an increased risk of several diseases, the consumption of low-fat dairy products can have positive health effects. Moreover, β-glucan is considered as a functional food ingredient, which also improves food’s textural properties. In this study, low-fat white-brined cheese was successfully manufactured by reducing 50% of the fat and by adding 0.4% β-glucan isolated from the mushroom *Pleurotus ostreatus*, in the form of paste. Generally, no differences were found between cheeses with or without the addition of β-glucan, regarding the biochemical properties, and no off-flavor or other defects were noted by the panelists. The addition of β-glucan, generally, improved white-brined cheeses’ textural properties. As β-glucan has already been used in other foods, such as yoghurts, biscuits, etc., the importance of these findings to the cheese industry is significant due to the great demand for good quality, low-fat, functional white-brined cheeses.

## Figures and Tables

**Table 1:** Quantity (%) of β-glucan in the white-brined cheese during ripening and storage ^(i)^.

Age (Days)	β-Glucan in Cheese (%)
1	0.75 ± 0.03 ^a^
15 ^(ii)^	0.37 ± 0.01 ^b^
30	0.41 ± 0.02 ^b^
60	0.44 ± 0.05 ^b^
90	0.39 ± 0.02 ^b^
120	0.44 ± 0.03 ^b^
180	0.42 ± 0.04 ^b^

^(i)^ Values are means of three cheese-making trials ± standard error. ^(ii)^ Day cheeses entered the cold room. Means of each parameter within the same column with different superscripts are significantly different (LSD test, *p* < 0.05).

**Table 2:** Physico-chemical characteristics of white-brined cheeses made with (G) or without (C) the addition of β-glucan, during ripening and storage ^(i)^.

Age (Days)	Type of Cheese	pH	Moisture%	Moisture in Non-Fat Substance %	Fat %	Fat in Dry Matter %
1	C	4.82 ± 0.04 ^a^	69.04 ± 0.03 ^a^	76.14 ± 0.17 ^a^	9.33 ± 0.17 ^a^	30.14 ± 0.57 ^a^
	G	4.80 ± 0.03 ^a^	70.68 ± 0.51 ^b^	77.38 ± 0.18 ^b^	8.67 ± 0.44 ^a^	29.55 ± 1.01 ^a^
15 ^(ii)^	C	4.36 ± 0.03 ^a^	63.31 ± 0.46 ^a^	72.02 ± 0.23 ^a^	12.10 ± 0.70 ^a^	32.94 ± 1.53 ^a^
	G	4.41 ± 0.03 ^a^	63.65 ± 1.17 ^a^	71.94 ± 0.87 ^a^	11.53 ± 0.93 ^a^	31.66 ± 1.89 ^a^
30	C	4.43 ± 0.02 ^a^	63.78 ± 0.13 ^a^	72.34 ± 0.30 ^a^	11.83 ± 0.33 ^a^	32.67 ± 0.91 ^a^
	G	4.41 ± 0.02 ^a^	64.45 ± 0.98 ^a^	72.62 ± 0.85 ^a^	11.27 ± 0.43 ^a^	31.67 ± 0.72 ^a^
60	C	4.43 ± 0.02 ^a^	64.78 ± 1.52 ^a^	72.99 ± 1.38 ^a^	11.27 ± 0.54 ^a^	32.01 ± 0.92 ^a^
	G	4.42 ± 0.03 ^a^	65.64 ± 0.26 ^a^	73.73 ± 0.18 ^a^	10.97 ± 0.17 ^a^	31.92 ± 0.29 ^a^
90	C	4.49 ± 0.03 ^a^	64.45 ± 0.13 ^a^	72.77 ± 0.33 ^a^	11.43 ± 0.30 ^a^	32.17 ± 0.90 ^a^
	G	4.51 ± 0.02 ^a^	65.68 ± 0.73 ^a^	73.33 ± 0.63 ^a^	10.43 ± 0.23 ^a^	30.40 ± 0.08 ^a^
120	C	4.43 ± 0.02 ^a^	64.65 ± 0.23 ^a^	73.16 ± 0.16 ^a^	11.63 ± 0.17 ^a^	32.90 ± 0.32 ^a^
	G	4.40 ± 0.01 ^a^	65.04 ± 0.41 ^a^	72.94 ± 0.32 ^a^	10.83 ± 0.33 ^a^	30.98 ± 0.73 ^a^
180	C	4.44 ± 0.02 ^a^	63.97 ± 0.36 ^a^	72.91 ± 0.13 ^a^	12.25 ± 0.50 ^a^	33.98 ± 1.08 ^a^
	G	4.43 ± 0.02 ^a^	65.79 ± 0.48 ^b^	74.06 ± 0.24 ^b^	11.17 ± 0.36 ^a^	32.62 ± 0.61 ^a^

^(i)^ Values are means of three cheese-making trials ± standard error. ^(ii)^ Day cheeses entered the cold room. Means of each parameter within the same column and day, with different superscripts, are significantly different (LSD test, *p* < 0.05).

**Table 3:** Physico-chemical characteristics of white-brined cheeses made with (G) or without (C) the addition of β-glucan, during ripening and storage ^(i)^.

Age (Days)	Type of Cheese	Salt %	Salt in Moisture %	Yield %	Acidity % Lactic Acid	aw	Ash %
1	C	N.M. ^(iii)^	N.M. ^(iii)^	28.98 ± 0.47 ^a^	1.88 ± 0.10 ^a^	0.981 ± 0.003 ^a^	1.90 ± 0.03 ^a^
	G			30.74 ± 0.78 ^a^	1.60 ± 0.03 ^b^	0.978 ± 0.004 ^a^	1.78 ± 0.03 ^a^
15 ^(ii)^	C	2.58 ± 0.11 ^a^	4.07 ± 0.14 ^a^	23.30 ± 0.45 ^a^	1.52 ± 0.05 ^a^	0.981 ± 0.002 ^a^	3.92 ± 0.05 ^a^
	G	2.44 ± 0.06 ^a^	3.84 ± 0.06 ^a^	24.32 ± 1.16 ^a^	1.41 ± 0.05 ^a^	0.979 ± 0.004 ^a^	3.85 ± 0.04 ^a^
30	C	3.13 ± 0.06 ^a^	4.91 ± 0.10 ^a^	24.18 ± 0.53 ^a^	1.24 ± 0.02 ^a^	0.959 ± 0.006 ^a^	4.11 ± 0.00 ^a^
	G	2.98 ± 0.14 ^a^	4.63 ± 0.23 ^a^	26.56 ± 0.48 ^b^	1.42 ± 0.08 ^a^	0.960 ± 0.007 ^a^	4.08 ± 0.06 ^a^
60	C	2.77 ± 0.03 ^a^	4.29 ± 0.14 ^a^	24.51 ± 0.56 ^a^	1.45 ± 0.04 ^a^	0.970 ± 0.004 ^a^	4.12 ± 0.06 ^a^
	G	2.72 ± 0.13 ^a^	4.15 ± 0.21 ^a^	25.85 ± 0.96 ^a^	1.38 ± 0.05 ^a^	0.970 ± 0.003 ^a^	4.08 ± 0.11 ^a^
90	C	2.39 ± 0.23 ^a^	3.70 ± 0.35 ^a^	24.61 ± 0.51 ^a^	1.19 ± 0.06 ^a^	0.977 ± 0.004 ^a^	4.10 ± 0.04 ^a^
	G	2.74 ± 0.25 ^a^	4.18 ± 0.41 ^a^	25.36 ± 0.63 ^a^	1.13 ± 0.05 ^a^	0.969 ± 0.003 ^a^	4.09 ± 0.09 ^a^
120	C	2.95 ± 0.08 ^a^	4.57 ± 0.11 ^a^	24.45 ± 0.14 ^a^	1.06 ± 0.02 ^a^	0.969 ± 0.003 ^a^	4.03 ± 0.06 ^a^
	G	2.90 ± 0.00 ^a^	4.46 ± 0.03 ^a^	25.65 ± 0.64 ^a^	1.13 ± 0.05 ^a^	0.967 ± 0.004 ^a^	3.96 ± 0.06 ^a^
180	C	3.00 ± 0.10 ^a^	4.69 ± 0.16 ^a^	23.78 ± 0.64 ^a^	1.10 ± 0.02 ^a^	0.980 ± 0.006 ^a^	3.87 ± 0.15 ^a^
	G	2.87 ± 0.09 ^a^	4.36 ± 0.11 ^a^	25.47 ± 1.19 ^a^	1.15 ± 0.04 ^a^	0.983 ± 0.007 ^a^	3.90 ± 0.06 ^a^

^(i)^ Values are means of three cheese-making trials ± standard error. ^(ii)^ Day cheeses entered the cold room. ^(iii)^ N.M.: Not Measured. Means of each parameter within the same column and day, with different superscripts, are significantly different (LSD test, *p* < 0.05).

**Table 4:** Textural characteristics of white-brined cheeses made with (G) or without (C) the addition of β-glucan, during storage ^(i)^.

Age (Days)	Type of Cheese	Hardness kg	Brittleness kg	Compression to Fracture %
60	C	39.68 ± 1.27 ^a^	16.40 ± 0.82 ^a^	18.80 ± 2.18 ^a^
	G	32.61 ± 1.57 ^b^	12.22 ± 0.70 ^b^	15.23 ± 0.80 ^a^
90	C	32.70 ± 0.48 ^a^	16.86 ± 0.12 ^a^	18.17 ± 1.01 ^a^
	G	27.59 ± 1.59 ^b^	11.28 ± 0.91 ^b^	15.24 ± 1.21 ^a^
120	C	27.99 ± 0.38 ^a^	19.51 ± 1.47 ^a^	22.75 ± 3.28 ^a^
	G	21.17 ± 1.25 ^b^	13.04 ± 1.28 ^b^	18.78 ± 0.67 ^a^
180	C	33.55 ± 2.24 ^a^	19.91 ± 2.13 ^a^	16.74 ± 1.72 ^a^
	G	30.71 ± 4.28 ^a^	15.20 ± 0.86 ^a^	16.93 ± 0.99 ^a^

^(i)^ Values are means of three cheese-making trials ± standard error. Means of each parameter within the same column and day, with different superscripts, are significantly different (LSD test, *p* < 0.05).

**Table 5:** Changes of color parameters of white-brined cheeses made with (G) or without (C) the addition of β-glucan during ripening and storage ^(i)^.

Age (Days)	Type of Cheese	Parameter L*	Parameter a*	Parameter b*
1	C	93.99 ± 0.34 ^a^	−8.91 ± 0.14 ^a^	8.94 ± 1.16 ^a^
	G	94.18 ± 0.68 ^a^	−8.53 ± 0.78 ^a^	8.00 ± 0.97 ^a^
15 ^(ii)^	C	93.64 ± 0.04 ^a^	−9.04 ± 0.51 ^a^	10.67 ± 0.24 ^a^
	G	93.74 ± 0.20 ^a^	−9.02 ± 0.61 ^a^	10.29 ± 0.09 ^a^
30	C	93.34 ± 0.28 ^a^	−10.33 ± 0.30 ^a^	10.46 ± 0.16 ^a^
	G	94.38 ± 0.98 ^a^	−9.75 ± 0.19 ^a^	10.20 ± 0.37 ^a^
60	C	95.54 ± 1.98 ^a^	−10.82 ± 0.51 ^a^	10.18 ± 0.26 ^a^
	G	92.89 ± 0.48 ^a^	−10.67 ± 0.52 ^a^	10.82 ± 0.56 ^a^
90	C	92.91 ± 0.49 ^a^	−14.03 ± 0.18 ^a^	10.73 ± 0.17 ^a^
	G	92.85 ± 0.21 ^a^	−14.01 ± 0.26 ^a^	10.71 ± 0.32 ^a^
120	C	93.59 ± 0.31 ^a^	−17.16 ± 0.09 ^a^	9.44 ± 0.28 ^a^
	G	93.63 ± 0.07 ^a^	−16.81 ± 0.06 ^b^	8.76 ± 0.67 ^a^
180	C	93.71 ± 0.21 ^a^	−16.65 ± 1.26 ^a^	9.65 ± 0.55 ^a^
	G	93.48 ± 0.14 ^a^	−16.43 ± 1.24 ^a^	9.80 ± 0.27 ^a^

^(i)^ Values are means of three cheese-making trials ± standard error. ^(ii)^ Day cheeses entered the cold room. Means of each parameter within the same column and day, with different superscripts, are significantly different (LSD test, *p* < 0.05).

**Table 6:** Organoleptic characteristics of mature white-brined cheeses made with (G) or without (C) the addition of β-glucan during storage ^(i)^.

Age (Days)	Type of Cheese	Appearance (10) ^(ii)^	Texture (40) ^(ii)^	Flavor (50) ^(ii)^	Total (100) ^(ii)^
60	C	8.9 ± 0.1 ^a^	33.4 ± 0.52 ^a^	42.5 ± 0.6 ^a^	84.9 ± 1.0 ^a^
	G	8.9 ± 0.1 ^a^	33.9 ± 0.47 ^a^	43.0 ± 0.9 ^a^	85.8 ± 1.2 ^a^
90	C	9.1 ± 0.1 ^a^	32.4 ± 0.40 ^a^	41.0 ± 0.6 ^a^	82.5 ± 0.7 ^a^
	G	9.1 ± 0.1 ^a^	34.3 ± 0.74 ^a^	38.8 ± 1.9 ^a^	82.2 ± 2.6 ^a^
120	C	9.0 ± 0.0 ^a^	32.93 ± 0.53 ^a^	42.3 ± 0.2 ^a^	84.3 ± 0.4 ^a^
	G	9.1 ± 0.1 ^a^	34.4 ± 0.61 ^a^	43.7 ± 0.4 ^b^	87.2 ± 1.1 ^a^
180	C	8.9 ± 0.1 ^a^	32.9 ± 0.58 ^a^	42.0 ± 0.5 ^a^	83.9 ± 1.0 ^a^
	G	9.1 ± 0.1 ^a^	32.4 ± 0.23 ^a^	43.3 ± 0.2 ^a^	86.8 ± 0.4 ^a^

^(i)^ Values are means of three cheese-making trials ± standard error. ^(ii)^ Values in brackets show the maximum scores. Means of each parameter within the same column and day, with different superscripts, are significantly different (LSD test, *p* < 0.05).

**Table 7:** Proteolysis of white-brined cheeses made with (G) or without (C) the addition of β-glucan during ripening and storage ^(i)^.

Age (Days)	Type of Cheese	TN, %	WSN %TN	TCA %TN	PTA %TN	Proteins %
1	C	2.99 ± 0.09 ^a^	6.61 ± 0.76 ^a^	3.17 ± 0.49 ^a^	0.53 ± 0.10 ^a^	19.07 ± 0.60 ^a^
	G	3.02 ± 0.06 ^a^	6.10 ± 0.33 ^a^	2.16 ± 0.10 ^a^	0.71 ± 0.05 ^a^	19.26 ± 0.38 ^a^
15 ^(ii)^	C	3.03 ± 0.04 ^a^	8.75 ± 0.81 ^a^	6.08 ± 0.36 ^a^	0.83 ± 0.26 ^a^	19.34 ± 0.25 ^a^
	G	2.93 ± 0.12 ^a^	9.08 ± 0.37 ^a^	6.05 ± 0.18 ^a^	0.68 ± 0.12 ^a^	18.71 ± 0.74 ^a^
30	C	2.87 ± 0.07 ^a^	8.66 ± 0.35 ^a^	5.95 ± 0.63 ^a^	0.63 ± 0.06 ^a^	18.31 ± 0.41 ^a^
	G	2.77 ± 0.05 ^a^	8.90 ± 0.55 ^a^	6.23 ± 0.89 ^a^	0.95 ± 0.21 ^a^	17.70 ± 0.29 ^a^
60	C	2.66 ± 0.05 ^a^	10.92 ± 0.92 ^a^	7.67 ± 0.62 ^a^	0.48 ± 0.10 ^a^	17.00 ± 0.29 ^a^
	G	2.74 ± 0.06 ^a^	10.49 ± 0.04 ^a^	7.68 ± 0.22 ^a^	1.29 ± 0.35 ^a^	17.51 ± 0.42 ^a^
90	C	2.77 ± 0.02 ^a^	11.17 ± 0.05 ^a^	7.23 ± 0.80 ^a^	1.11 ± 0.12 ^a^	17.70 ± 0.13 ^a^
	G	2.67 ± 0.06 ^a^	9.41 ± 0.40 ^b^	6.63 ± 0.20 ^a^	0.90 ± 0.05 ^a^	17.01 ± 0.38 ^a^
120	C	2.84 ± 0.04 ^a^	7.44 ± 0.67 ^a^	5.62 ± 0.41 ^a^	0.65 ± 0.12 ^a^	18.12 ± 0.27 ^a^
	G	2.62 ± 0.00 ^b^	8.91 ± 0.43 ^a^	7.01 ± 0.32 ^a^	0.94 ± 0.10 ^a^	16.72 ± 0.02 ^b^
180	C	2.86 ± 0.03 ^a^	11.01 ± 0.21 ^a^	8.58 ± 0.11 ^a^	1.08 ± 0.12 ^a^	18.22 ± 0.18 ^a^
	G	2.63 ± 0.10 ^a^	12.27 ± 0.78 ^a^	9.37 ± 0.59 ^a^	1.12 ± 0.22 ^a^	16.79 ± 0.62 ^a^

^(i)^ Values are means of three cheese-making trials ± standard error. ^(ii)^ Day cheeses entered the cold room. Means of each parameter within the same column and day, with different superscripts, are significantly different (LSD test, *p* < 0.05).

**Table 8:** Free fatty acids content (μg/g) of white-brined cheeses made with (G) or without (C) the addition of β-glucan during ripening and storage ^(i)^.

Free Fatty Acids	1 Day		15 Day ^(ii)^		30 Day	
	C	G	C	G	C	G
C2	25.9 ± 1.67 ^a^	33.0 ± 3.90 ^a^	60.6 ± 9.31 ^a^	63.5 ± 5.65 ^a^	55.0 ± 4.24 ^a^	69.69 ± 0.29 ^a^
C4	1.85 ± 0.5 ^a^	1.62 ± 0.1 ^a^	8.06 ± 1.04 ^a^	4.26 ± 0.21 ^a^	7.18 ± 0.06 ^a^	7.87 ± 0.02 ^a^
C4:iso	1.21 ± 0.2 ^a^	2.50 ± 0.1 ^a^	2.69 ± 0.15 ^a^	2.87 ± 0.06 ^a^	2.18 ± 0.3 ^a^	2.78 ± 0.4 ^a^
C5:iso	0.83 ± 0.3 ^a^	1.39 ± 0.8 ^a^	1.44 ± 0.06 ^a^	1.39 ± 0.2 ^a^	2.22 ± 0.2 ^a^	1.99 ± 0.1 ^a^
C6	2.66 ± 1.72 ^a^	3.71 ± 1.69 ^a^	3.10 ± 1.12 ^a^	4.56 ± 0.61 ^a^	2.70 ± 0.18 ^a^	3.92 ± 0.4 ^a^
C8	25.55 ± 1.31 ^a^	30.77 ± 1.17 ^a^	27.49 ± 0.23 ^a^	27.08 ± 0.3 ^a^	27.35 ± 0.5 ^a^	30.05 ± 1.25 ^a^
C10	13.85 ± 0.47 ^b^	5.05 ± 3.18 ^a^	448.0 ± 17.47 ^a^	382 ± 26.42 ^a^	412.0 ± 8.87 ^a^	394.0 ± 6.67 ^a^
C12	49.95 ± 3.85 ^a^	61.4 ± 3.31 ^b^	57.6 ± 2.53 ^a^	61.25 ± 3.31 ^a^	60.56 ± 2.07 ^a^	57.35 ± 1.98 ^a^
C14	44.5 ± 2.61 ^b^	19.7 ± 0.6 ^a^	52.8 ± 2.74 ^a^	64.2 ± 0.79 ^a^	59.8 ± 4.09 ^a^	103.45 ± 2.12 ^a^
C16	98.8 ± 4.41 ^b^	62.45 ± 2.27 ^a^	123.0 ± 3.44 ^a^	164.0 ± 0.37 ^b^	111.0 ± 7.92 ^a^	161.0 ± 3.13 ^b^
C18	54.15 ± 5.39 ^b^	37.0 ± 0.88 ^a^	54.25 ± 6.69 ^a^	38.86 ± 3.95 ^a^	58.6 ± 2.5 ^a^	57.72 ± 0.14 ^a^
C18:1	70.90 ± 6.35 ^b^	19.65 ± 3.06 ^a^	88.25 ± 8.40 ^b^	35.35 ± 5.76 ^a^	60.9 ± 7.62 ^a^	49.45 ± 5.03 ^a^
C18:2	22.35 ± 2.37 ^a^	17.32 ± 1.2 ^a^	27.25 ± 1.8 ^a^	27.06 ± 0.84 ^a^	23.7 ± 0.97 ^a^	25.70 ± 0.84 ^a^
Total free fatty acids	412.5 ± 11.61 ^a^	295.6 ± 1.29 ^a^	954.5 ± 22.16 ^a^	876.4 ± 14.62 ^a^	883.2 ± 84.46 ^a^	965 ± 1.5 ^a^

^(i)^ Values are means of three cheese-making trials ± standard error. ^(ii)^ Day cheeses entered the cold room. Means of each parameter within the same row and day, with different superscripts, are significantly different (LSD test, *p* < 0.05).

**Table 9:** Free fatty acids content (μg/g) of white-brined cheeses made with (G) or without (C) the addition of β-glucan during ripening and storage ^(i)^.

Free Fatty Acids	60 Day		90 Day		120 Day		180 Day	
	C	G	C	G	C	G	C	G
C2	57.13 ± 3.08 ^a^	73.94 ± 5.17 ^a^	60.01 ± 1.22 ^a^	55.37 ± 1.81 ^a^	77.83 ± 2.61 ^a^	65.28 ± 2.47 ^a^	57.22 ± 2.6 ^a^	110,0 ± 7.2 ^b^
C4	9.45 ± 0.31 ^a^	9.08 ± 2.68 ^a^	8.30 ± 1.78 ^a^	13,0 ± 1.25 ^a^	12.92 ± 1.65 ^a^	11.25 ± 1.94 ^a^	10.09 ± 3.2 ^a^	14.91 ± 3.63 ^a^
C4:iso	2.5 ± 0.09 ^a^	4.44 ± 1.11 ^a^	2.30 ± 0.03 ^a^	2.91 ± 0.01 ^a^	3.38 ± 0.11 ^a^	3.15 ± 0.16 ^a^	2.64 ± 0.2 ^a^	3.89 ± 0.5 ^a^
C5:iso	4.26 ± 0.72 ^a^	2.65 ± 0.09 ^a^	3.18 ± 0.38 ^a^	2.79 ± 0.09 ^a^	1.44 ± 0.11 ^a^	2.87 ± 0.62 ^a^	1.07 ± 0.26 ^a^	2.23 ± 0.1 ^a^
C6	3.9 ± 0.43 ^a^	4.64 ± 0.61 ^a^	4.45 ± 1.69 ^a^	4.79 ± 0.47 ^a^	5.21 ± 0.21 ^a^	4.30 ± 0.42 ^a^	9.76 ± 2.8 ^a^	5.34 ± 0.1 ^a^
C8	27.81 ± 0.04 ^a^	27.49 ± 0.07 ^a^	26.85 ± 0.04 ^a^	27.08 ± 0.1 ^a^	27.57 ± 0.1 ^a^	27.99 ± 0.23 ^a^	27.51 ± 0.5 ^a^	27.43 ± 0.4 ^a^
C10	516.0 ± 2.5 ^a^	412.0 ± 2.95 ^a^	449,90 ± 19.9 ^a^	517.0 ± 13.7 ^a^	664.0 ± 22.91 ^b^	555.0 ± 21.44 ^a^	680.0 ± 11.8 ^a^	745.0 ± 26.32 ^a^
C12	61.35 ± 2.13 ^a^	69.55 ± 1.07 ^a^	58.97 ± 4.41 ^a^	70.31 ± 8.07 ^a^	68.75 ± 1.91 ^a^	65.33 ± 2.56 ^a^	85.75 ± 1.37 ^a^	84.4 ± 2.14 ^a^
C14	51.5 ± 3.16 ^a^	54.85 ± 2.45 ^a^	65.43 ± 3.07 ^a^	75.8 ± 2.15 ^a^	77.9 ± 3.19 ^a^	63.82 ± 0.23 ^a^	60.37 ± 4.32 ^a^	57.10 ± 4.38 ^a^
C16	113.0 ± 5.91 ^a^	116.0 ± 4.47 ^a^	139.60 ± 1.06 ^a^	142.0 ± 7.67 ^a^	163.0 ± 8.82 ^a^	135.0 ± 1.26 ^a^	132.0 ± 9.7 ^a^	134.0 ± 8.52 ^a^
C18	62.15 ± 1.8 ^a^	55.65 ± 2.16 ^a^	68.57 ± 3.85 ^a^	58.74 ± 5.89 ^a^	80.0 ± 6.4 ^a^	60.47 ± 3.84 ^a^	121.95 ± 7.33 ^a^	98.95 ± 8.09 ^a^
C18:1	60.7 ± 8.33 ^a^	63.85 ± 7.46 ^a^	61.93 ± 5.56 ^a^	71.17 ± 6.9 ^a^	89.95 ± 9.8 ^a^	71.96 ± 0.38 ^a^	67.70 ± 1.27 ^a^	61.40 ± 2.94 ^a^
C18:2	23.85 ± 2.92 ^a^	47.0 ± 4.94 ^b^	39.45 ± 3.29 ^a^	49.69 ± 2.15 ^a^	81.72 ± 0.07 ^a^	79.65 ± 0.24 ^a^	57.85 ± 4.30 ^a^	87.85 ± 10.21 ^a^
Total free fatty acids	994 ± 19.46 ^a^	941 ± 11.18 ^a^	989 ± 31.98 ^a^	1061 ± 11.01 ^a^	1354 ± 6.65 ^a^	1146 ± 19.13 ^a^	1314 ± 48.63 ^a^	1433 ± 39.53 ^b^

^(i)^ Values are means of three cheese-making trials ± standard error. Means of each parameter within the same row and day, with different superscripts, are significantly different (LSD test, *p* < 0.05).
